# Boronate-Based Inhibitors of Penicillin-Binding Proteins: An Underestimated Avenue for Antibiotic Discovery?

**DOI:** 10.3390/ph18091325

**Published:** 2025-09-04

**Authors:** Valentina Villamil, Luca Svolacchia Brusoni, Fabio Prati, Emilia Caselli, Nicolò Santi

**Affiliations:** Department of Life Sciences, Università degli Studi di Modena e Reggio Emilia (UNIMORE), Via Campi 103, 41125 Modena, Italy; vivalent@unimore.it (V.V.); l.svolacchia@unimore.it (L.S.B.); fabio.prati@unimore.it (F.P.); emilia.caselli@unimore.it (E.C.)

**Keywords:** antimicrobial resistance, penicillin-binding proteins, boron-based inhibitors, boronic acid inhibitors, boronic acids

## Abstract

Penicillin-binding proteins (PBPs) are essential enzymes involved in bacterial cell wall biosynthesis and represent the primary targets of β-lactam antibiotics. However, the efficacy of these agents is threatened by β-lactamase production and PBP alterations, prompting the search for alternative strategies. In this context, boronic acids, long established as potent inhibitors of serine β-lactamases (SBLs), have been proposed as scaffolds for PBP inhibition based on the shared structural and mechanistic features of these enzyme families. This perspective provides a literature-based survey with structural analysis to evaluate emerging evidence on the potential role of boronic acids as PBP-targeting agents, with a particular focus on peptidomimetic boronic acids, repurposed β-lactamase inhibitors, and novel scaffold architectures. While early work showed limited activity against low-molecular-mass PBPs, more recent compounds, particularly certain bicyclic boronates, have demonstrated potent binding and, in some cases, antibacterial activity. Structural analyses reveal diverse binding modes and underscore the role of conformational dynamics in modulating affinity. Despite these advances, significant challenges remain, including target selectivity, membrane permeability, and species-specific differences. Nevertheless, the direct inhibition of PBPs by boronic acids, while still in early development, may offer a viable complement or alternative to β-lactam therapy, warranting further exploration through structure-guided design and comprehensive biological evaluation. Here, we analyze the potential of boronic acid inhibitors (BAIs) to target PBP enzymes, considering their promise as non-β-lactam antimicrobial agents with possible clinical relevance.

## 1. Introduction

β-lactam antibiotics (BLAs) are frontline treatments for bacterial infections due to their broad-spectrum activity, well-established efficacy, and safety. Their bactericidal action results from inhibiting bacterial cell wall synthesis by covalently binding to the transpeptidase domain of penicillin-binding proteins (PBPs) [1,2]. Specifically, PBPs catalyze the final steps of peptidoglycan (PG) biosynthesis by mediating the cross-linking of short peptide units within the growing matrix through coordinated glycosyltransferase (GT) and transpeptidase (TP) activities [3]. PG represents an excellent antibacterial target due to its essential role in maintaining bacterial cell wall integrity. As a highly conserved and bacteria-specific structure, its inhibition compromises cell wall stability, leading to cell lysis, making it an ideal point of intervention for antimicrobial therapies.

PBPs are classified into two main categories: high-molecular-mass (HMM) PBPs and low-molecular-mass (LMM) PBPs. HMM PBPs are subdivided into two classes, depending on their structure and catalytic activity. Class A are multifunctional enzymes with both GT and TP activity, essential for PG synthesis (e.g., PBP1a and PBP1b in *E. coli*). Class B are monofunctional, performing only transpeptidation (e.g., PBP2 and PBP3 in *E. coli*). LMM PBPs are frequently described with the general term of class C PBPs, sometimes with C1, C2, and C3 as subdivisions, or alternatively as class A, B, and C within the LMM category. These enzymes act mainly as carboxypeptidases or endopeptidases, therefore performing generally non-essential activities, and they are implicated in cell wall maintenance and stress adaptation (e.g., PBP4–PBP7 in *E. coli*) [4].

**Figure 1:**
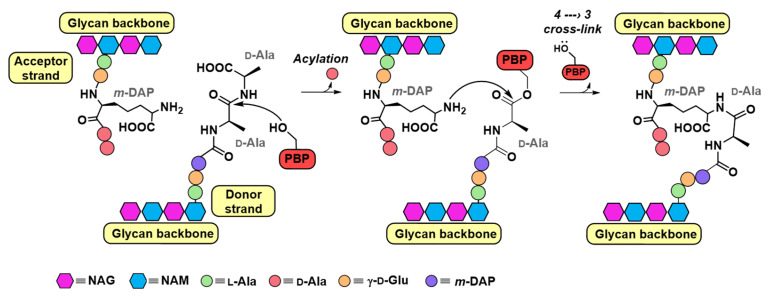
Mechanism of PBP transpeptidases. The PBP nucleophilic serine attacks a pentapeptide on the donor strand, forming a peptide–enzyme complex and releasing d-alanine. From the acceptor strand of peptidoglycan, a nucleophilic side chain (here, *m*-DAP) reacts with the peptide–enzyme complex, forming a 4 → 3 peptidoglycan cross-link, followed by release of the enzyme.

The catalytic mechanism mediated by PBPs involves initial glycan strand synthesis via GT activity, followed by the TP-catalyzed cross-linking of the pentapeptide chains within the PG matrix through a two-step process. First, the active-site serine attacks the d-Ala-d-Ala bond of a donor pentapeptide (e.g., l-Ala-γ-d-Glu-*m*-DAP-d-Ala-d-Ala in *E. coli*), forming a covalent acyl–enzyme intermediate (Figure 1). Subsequently, the ε-amino group of the *m*-DAP residue from an acceptor strand performs a nucleophilic attack on this intermediate, forming a new amide bond and generating 4 → 3 cross-links [5,6]. These cross-links are crucial for PG structural integrity. BLAs, bearing a conserved β-lactam core, irreversibly inhibit the TP domains by mimicking the natural substrate acyl-d-Ala-d-Ala [7]. The canonical active site serine attacks the β-lactam ring, forming a stable acyl–enzyme complex, resembling the first step of the native transpeptidation reaction, and leading to prolonged inhibition of TP activity, followed by PG cross-linking disruption and bacterial cell death [4]. BLAs efficiently target all classes of PBPs; therefore, in vitro studies of β-lactam–PBP interactions often employ LMM PBPs, which are easier to express, purify, and assay, while retaining sufficient enzymatic activity to accurately assess binding kinetics. However, the antimicrobial activity of β-lactams depends on the inactivation of at least one HMM PBP, highlighting the need for caution when extrapolating findings from LMM PBPs to overall antibiotic efficacy [5].

Over the past decades, extensive studies have focused on the structural modifications of BLAs (particularly to side chains and ring systems) to enhance pharmacokinetic properties, broaden the antibacterial spectrum of activity, and improve stability [8]. Although certain non-β-lactam agents (e.g., vancomycin and dalbavancin) interfere with cell wall biosynthesis by targeting PG precursors, no alternative scaffold has demonstrated clinically validated efficacy in targeting the TP domain of PBPs [9]. Consequently, the β-lactam ring remains the only pharmacophore successfully exploited for direct PBP inhibition in clinical settings [10]. However, bacteria have progressively developed various resistance mechanisms. In Gram-positive bacteria, a prominent mechanism of resistance to BLA involves structural modifications of the PBPs, giving rise to enzyme variants with reduced affinity for BLAs [5]. This phenomenon is well documented in many clinically important pathogens such as *Streptococcus pneumoniae* [11] and *Neisseria* spp. [12]. Furthermore, methicillin-resistant *Staphylococcus aureus* (MRSA) exemplifies a distinct mechanism of β-lactam resistance compared with typical Gram-positive bacteria. Instead of modifying existing PBPs, MRSA has acquired a novel transpeptidase, PBP2a, encoded by the *mecA* gene. PBP2a exhibits intrinsically low affinity for β-lactam antibiotics, allowing the bacteria to continue peptidoglycan cross-linking even when the native PBPs are inhibited. This resistance is largely due to the structural inaccessibility of the enzyme active site, which limits β-lactam acylation and enables MRSA to sustain cell wall synthesis under antibiotic pressure [13].

Still, the production of β-lactamases (BLs) remains the most widespread and clinically relevant means of resistance to BLAs, particularly in Gram-negative bacteria [14]. These enzymes mediate the hydrolysis of the β-lactam ring, thereby inactivating the compound before it can exert its antibacterial effect [5]. Structural and mechanistic similarities highlight a common evolutionary origin between serine-β-lactamases (SBLs) and PBPs [4]. SBLs likely arose from ancestral PBPs via divergent evolution, adapting their enzymatic machinery from cell wall biosynthesis to antibiotic inactivation by exploiting the structural mimicry between BLA and the d-Ala-d-Ala dipeptide motif [15]. Clinically, the most successful strategy to overcome BL-mediated resistance involves the co-administration of appropriate BLAs with β-lactamase inhibitors (BLIs), which inactivate the enzymatic activity of BLs and restore the antibiotic ability to reach and inhibit its PBP target [16]. BLIs are broadly classified into β-lactam-based and non-β-lactam-based inhibitors. Traditional β-lactam-based, irreversible covalent inhibitors (e.g., clavulanic acid [17], sulbactam [18], tazobactam [19], and, recently, enmetazobactam [20]) mainly target class A SBL but have limited activity against classes B (metallo-β-lactamases (MBLs)), C, and D and are vulnerable to degradation by multidrug-resistant (MDR) pathogens overexpressing BLs. To address these limitations, diazabicyclooctanes (DBOs) have emerged as a novel class of non-β-lactam-based, covalent reversible inhibitors (e.g., avibactam [21], relebactam [22], and durlobactam [23]), targeting class A and C SBLs.

Although DBOs offer a promising alternative to β-lactam-based compounds, due to their reduced susceptibility to hydrolysis by BLs, they generally exhibit limited efficacy against most class D SBLs and, more critically, MBLs. The emergence of clinically relevant bacterial strains co-expressing both SBLs and MBLs has prompted the development of novel treatment strategies to address resistance mediated by both enzyme families. Current therapeutic options include combinations of aztreonam, which is stable against MBL-mediated hydrolysis, plus ceftazidime/avibactam to inhibit co-produced SBLs [24]. While effective, these combinations introduce complexity into clinical management and may not always be practical or accessible. Consequently, emerging strategies to combat β-lactamase-mediated resistance focus on the development of novel cross-class β-lactamase inhibitors, single-molecule agents capable of targeting both SBLs and MBLs. In this context, boronic acid inhibitors (BAIs) have arisen as a novel class of reversible covalent inhibitors. Despite being chemically different from BLAs and other classes of BLIs, such as DBO, they can achieve the simultaneous inhibition of mechanistically distinct SBL and MBL enzymes, enhancing their stability and therapeutic potential. Unlike β-lactam antibiotics, which are readily inactivated by nucleophilic attack from catalytic residues within β-lactamases, BAIs lack the strained four-membered β-lactam ring that constitutes the primary recognition and cleavage site, rendering them inherently less prone than naturally occurring β-lactams to elicit resistance. However, it remains conceivable that bacterial resistance mechanisms could eventually arise against clinically employed boronic acids. Notably, aside from associated porin mutations and overexpression of efflux pumps observed for almost all antibacterial agents, no significant resistance to boronic acid antibacterials has been reported yet [25,26,27].

**Figure 2:**
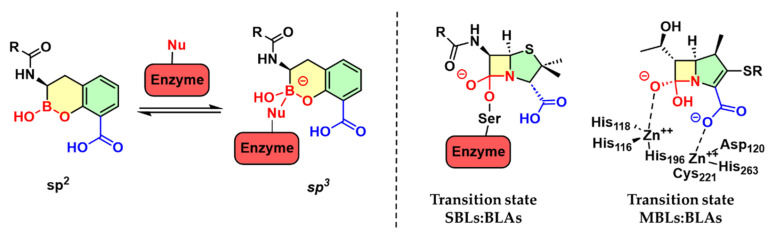
Boronic acid transition state inhibitors, such as SBL and MBL inhibitors. The boron atom changes the hybridization from sp^2^ to sp^3^ and mimics the high-energy anionic tetrahedral transition state formed during the catalysis of both enzyme families.

The mechanism by which BAIs achieve SBL inhibition is well understood and involves the formation of an enzyme–inhibitor complex, where the boron atom forms a reversible covalent bond with the catalytic serine residue in the active site, changing the hybridization from sp^2^ to sp^3^ and mimicking the high-energy anionic tetrahedral transition state formed during catalysis (Figure 2) [25]. The clinical advancement of boronic acid derivatives as BL inhibitors highlights their significant therapeutic potential. Vaborbactam, effective against class A and C SBLs, is approved in combination with meropenem for treating carbapenem-resistant *Enterobacteriaceae* infections, notably complicated urinary tract infections [28,29]. Recently, bicyclic boronates such as taniborbactam [30] and xeruborbactam [31] have demonstrated broad-spectrum inhibition of both SBLs and MBLs and have progressed through phase 3 and phase 1 clinical trials, respectively.

Although BLIs were initially developed as an adjuvant to BLAs, without inherent antibiotic activity, several have demonstrated affinity for various PBPs. Notably, sulbactam [32,33] (a β-lactam-based inhibitor), avibactam [34], and zidebactam [32,35] (non-β-lactam DBOs) all display inhibitory activity against PBPs. At this point, some important considerations merit emphasis: (i) BLAs interact with both PBPs and SBLs, acting as covalent inhibitors of the former and as hydrolysable substrates of the latter, reflecting the evolutionary and mechanistic relationship between these enzyme families; (ii) several BLIs, designed to inhibit SBLs, have also demonstrated PBP inhibition, underscoring the potential of a single-chemical scaffold to simultaneously inhibit evolutionarily related targets; and (iii) likewise, BAIs are well-established substrates and inhibitors of SBLs, in particular, the cyclic scaffolds are cross-class inhibitors. In light of this parallel, it is reasonable to hypothesize that boronic acid inhibitors, analogously to BLAs, might also interact with and inhibit PBPs [36].

PBPs have been historically underestimated as targets for boronic acid inhibitors, as research has primarily focused on β-lactamases due to their direct role in resistance and the assumption that PBPs were already addressed by classical β-lactams. Challenges such as structural complexity, limited SAR data, technical difficulties in screening assays, and bacterial permeability barriers have further contributed to this perception. Nevertheless, BAIs offer unique advantages, including intrinsic antibacterial activity, tunable selectivity, and the ability to circumvent common resistance mechanisms. By directly targeting PBPs as the primary focus, rather than β-lactamases, BAIs can provide a more straightforward and effective therapeutic strategy, holding promise as a novel generation method for antibacterial agents, enhancing clinical utility. Accordingly, the following sections explore the therapeutic potential of these compounds, highlighting their promise as non-β-lactam antimicrobial agents.

## 2. Discussion

### 2.1. Strategies for Designing Boronic Acid-Based PBP Inhibitors

**Figure 3:**
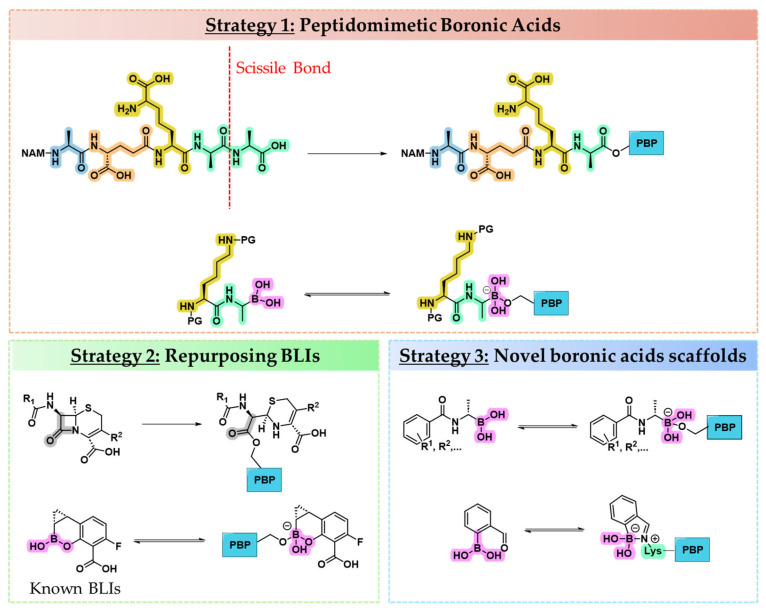
**Strategy 1.** **Top**: an explanatory example of an *E. coli* PBP substrate peptide and its relative acyl–PBP product. Highlighted in blue is l-Ala, in orange γ-d-Glu, in yellow *m*-DAP (*meso*-diaminopimelic acid), and in green d-Ala. **Bottom**: an explanatory example of a peptide boronic acid transition state analog and its relative acyl–enzyme intermediate. **Strategy 2.** **Top**: an explanatory example of a cephalosporin and its relative acyl–PBP product. The carbonyl group is highlighted in grey. **Bottom**: known boron-based BLIs repurposed as PBP inhibitors. The boronic acid is highlighted in fuchsia. **Strategy 3:** an explanatory example of novel boron-based compounds designed for PBP inhibition and relative complexes in the presence of PBPs.

Several strategies have been developed to identify the most promising boronic acid inhibitors of PBPs. One rational approach is substrate mimicry, in which a known stem peptide sequence, the natural substrate of PBPs, is used as a template, with the scissile bond replaced by a boronic acid moiety to mimic the tetrahedral transition state (Figure 3, Strategy 1) [37]. While this approach is grounded in strong biological logic, it faces three key limitations: (i) stem peptide sequences differ across bacterial species and among various PBPs, reducing broad-spectrum applicability [4]; (ii) the synthesis of peptidomimetic structures might be complex and low-yielding, posing practical barriers to large-scale optimization [38]; and (iii) the high molecular weight and the polar nature of these compounds might sensibly reduce their cell membrane permeability.

A second, more pragmatic strategy draws inspiration from BLIs, many of which integrate the boronic acid pharmacophore within side chains derived from BLAs (Figure 3, Strategy 2). Repurposing boron-based BLIs to PBPs leverages the structural similarity between SBLs and PBPs but may fail to account for the unique substrate recognition features and mechanistic nuances of PBPs [39].

A third, exploratory avenue focuses on the design and identification of novel scaffolds intrinsically compatible with boronic acid incorporation (Figure 3, Strategy 3). This approach emphasizes structure-based drug design, informed by structure–activity relationships (SARs), crystallography, and computational modeling. It allows for the tailoring of molecular geometry, electronic properties, and side chain orientation to selectively engage PBP active sites, offering a promising but less charted path toward next-generation PBP inhibitors [40].

#### 2.1.1. Peptidomimetic Boronic Acid Inhibitors

**Figure 4:**
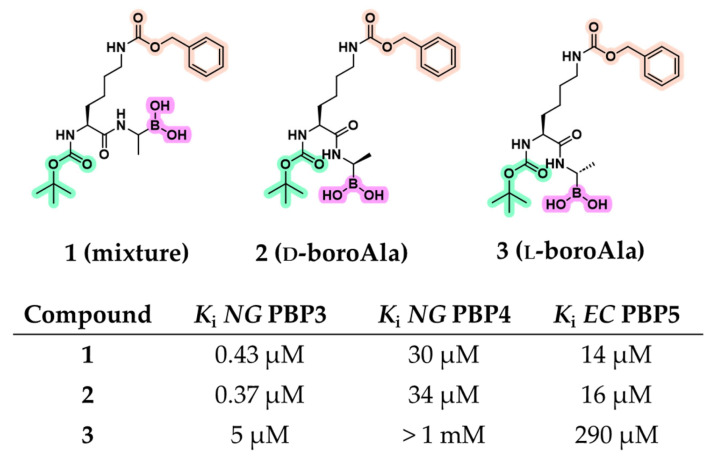
**Top**: structures of the peptide boronic acids reported by Pechenov et al. Highlighted in green is the BOC group, in pink the CBZ group, and in fuchsia the boronic acids. **Bottom**: affinity data for compounds **1**–**3** with LMM PBPs.

Early peptidomimetic boronic acid inhibitors were initially used as probes to study the enzymology of PBPs, exploiting substrate mimicry to investigate catalytic mechanisms, substrate specificity, and SAR analysis. In a landmark study, Pechenov et al. synthesized several transition-state analogs by modifying natural peptidyl substrates with electrophilic groups (e.g., aldehydes, trifluoromethyl ketones, chloromethyl ketones, and boronic acids) capable of covalent interaction with active-site serine [36]. The dipeptide Boc-l-Lys(Cbz)-d-Ala was selected as a scaffold, mimicking a portion of the PBP substrate up to the cleavage site. A panel of LMM PBPs with measurable enzymatic activity against d-Ala-d-Ala-based substrates, including *Escherichia coli* (*EC*) PBP5 (class A) and *Neisseria gonorrhoeae* (*NG*) PBPs 3 and 4 (class C), was used for biological evaluation. Although LMM PBPs are not essential for bacterial viability and are not primary targets of β-lactams, their active site architecture is highly conserved, making them suitable models for probing PBP enzymology. Among seven tested inhibitors, peptide boronic acids were the most effective, demonstrating inhibitory activity against all three tested PBPs, an outcome not observed with the other compound classes (Figure 4). Initially, compound **1**, synthesized as a pinacol-protected diastereomeric mixture of peptide boronic acids derived from d- and l-boroalanine, was evaluated. It exhibited a potent inhibition of *NG* PBP3 (*K*_i_ = 0.43 μM), along with moderate affinity for *NG* PBP4 (*K*_i_ = 30 μM) and *EC* PBP5 (*K*_i_ = 14 μM). To discern the active diastereoisomer, compounds **2** (d-boroAla) and **3** (l-boroAla) were obtained using (−)- and (+)-pinanediol as a chiral auxiliary. Compound **2** retained strong inhibitory activity, slightly improving the *K*_i_ for *NG* PBP3 observed with compound **1**, while maintaining comparable activity against *NG* PBP4 and *EC* PBP5. Kinetic analyses confirmed that compound **2** acts as a competitive inhibitor, engaging the catalytic serine within the active site [36].

**Figure 5:**
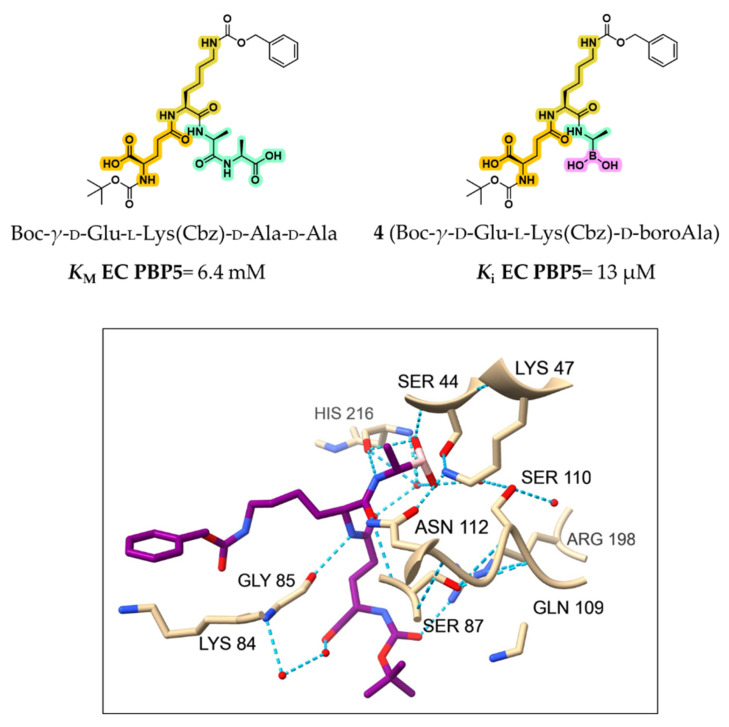
**Top**: structures and activities for *EC* PBP5 for the model peptide and compound **4**. Highlighted in orange is γ-d-Glu, in yellow l-Lys, in green d-Ala, and in fuchsia the boronic acid portion. **Bottom**: crystal structure of the *EC* PBP5:**4** complex (PDB: 1Z6F). Compound **4** is colored in purple, while the light blue dashed lines represent H-bonds.

The same group further investigated compound **4** (Boc-γ-d-Glu-l-Lys(Cbz)-d-boroAla), a boronic acid designed to mimic the substrate fragment remaining after acylation and loss of the terminal d-Ala [41]. Compound **4** displayed a *K*_i_ value of 13 μM, markedly lower than the *K*_M_ of the parent substrate Boc-γ-d-Glu-l-Lys(Cbz)-d-Ala-d-Ala (*K*_M_ = 6.4 mM), underscoring the advantage of transition-state mimicry (Figure 5).

Analysis of the *EC* PBP5–compound **4** complex (PDB: 1Z6F) confirmed the covalent bonding between boron and Ser44, along with a stabilizing hydrogen bond network (see Figure 5). The boron-bound hydroxyls interacted with His216 and two structural waters, while the adjacent amide NH formed a hydrogen bond with the backbone of His216. The amide carbonyl further interacted with Ser87 and Asn112, contributing to binding affinity. These interactions collectively stabilized compound **4** within the active site and explained its inhibitory potency. However, the poor resolution of flexible moieties suggested that larger or extended groups may fail to form productive contacts. This case clearly illustrates both the potential and limitations of early boronic acid-based PBP inhibitors and the need for more stable, structurally refined analogs [41].

**Figure 6:**
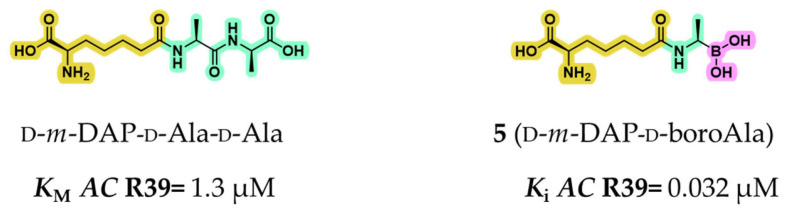
Structures and activity against *AC* R39 for the peptide d-*m*-DAP-d-Ala-d-Ala and compound **5**. Highlighted in yellow l-Lys, in green d-Ala, and in fuchsia the boronic acid portion.

Following the identification of tripeptide boronic acid **4**, a limited number of studies further employed peptidomimetic boronic acids to examine the catalytic mechanisms and substrate preferences of PBPs. For example, Dzhekieva et al. developed compound **5**, (d-α-aminopimelylamino)-d-1-ethylboronic acid, a peptidomimetic featuring a d-*m*-DAP–d-Ala motif, a portion mimicking the core peptidoglycan fragment. Compound **5** exhibited a *K*_i_ value of 32 nM against the LMM d,d-peptidase *Actinomadura* (*AC)* R39, representing nearly 100-fold greater affinity than its natural tripeptide analog d-*m*-DAP–d-Ala–d-Ala (*K*_M_ = 1.3 μM; Figure 6) [42]. These promising results were further reinforced by the crystal structure of *AC* R39 bound to compound **5** (PDB: 2XDM), which revealed key interactions underpinning its potency, such as a hydrophobic channel formed by Met414 and Tyr147, which accommodates the tetramethylene side chain through van der Waals contacts [42].

**Figure 7:**
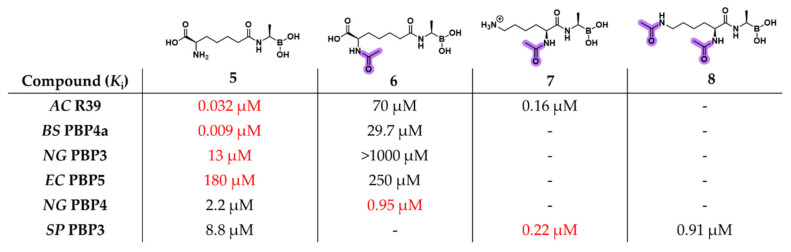
Structures of the peptidomimetic boronic acids **5**–**8** and their relative activity (*K*_i_) for a panel of LMM PBPs. In violet is highlighted the acetyl protecting group. Within the table, the red writing indicates the best inhibitor for each PBP.

To deepen the understanding of PBP substrate preferences, Nemmara et al. systematically evaluated a series of peptidoglycan-mimetic peptide substrates and boronic acid inhibitors across several LMM PBPs, including *EC* PBP5, *NG* PBPs 3 and 4, *Bacillus subtilis* (*BS*) PBP4a, *Streptococcus pneumoniae* (*SP*) PBP3, and *AC* R39 [43]. Their study aimed to investigate how *N*-terminal acylation (compounds **6**–**8**) influenced specificity (Figure 7). Boronic acid derivatives once again consistently showed tighter binding and superior inhibition compared with unmodified peptide substrates, highlighting their utility in probing the enzymatic landscape of PBPs.

Eventually, Dzhekieva et al. examined the activity of several peptidoglycan-mimetic boronic acids, including compounds **5** and **6**, against LMM and HMM PBPs from *E. coli* [37]. On the one hand, these inhibitors successfully bound to LMM PBPs in membrane preparations and whole cells. On the other hand, peptidomimetic boronic acids displayed no detectable interaction with HMM enzymes. Thus, authors concluded that peptidomimetic boronic acids fail to induce stable, high-affinity binding in HMM PBPs under physiological conditions. Although originally conceived as mechanistic probes, the potent in vitro inhibition of multiple PBP isoforms prompted reconsideration of these compounds as potential antibiotic leads. However, their development has stalled, largely due to limited in vivo efficacy and insufficient engagement of essential HMM PBPs, which is the therapeutic goal. The rather poor in vivo efficacy of peptidomimetic boronic acids can largely be attributed to pharmacological constraints. Their large size and high polarity, reminiscent of therapeutic peptides, severely restrict cell envelope penetration and result in poor in vivo stability, potentially resulting in rapid degradation and short half-lives. As a consequence, these compounds showed potent enzyme inhibition but did not achieve antibacterial activity, as reflected by the absence of MIC data. In contrast, more compact and chemically diverse boronic acid subclasses have successfully advanced as BLIs and, as further discussed in the next sections, hold potential as PBP inhibitors [44].

#### 2.1.2. Repurposing Known BLIs as PBP Inhibitors

**Figure 8:**
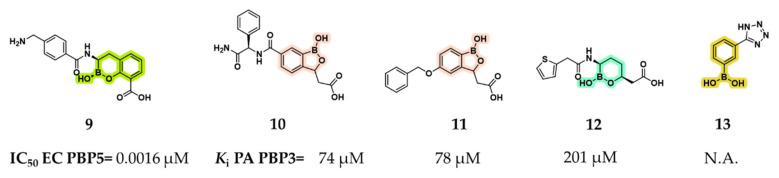
Structures of compounds **9**–**13** and their relative activities for *EC* PBP5 (compound **9**) and *PA* PBP3 (compounds **10**–**13**). Benzoxaborinine rings are highlighted in green, benzoxaboroles in pink, monocyclic boronates in cyan, and phenylboronic acids in yellow.

A second approach for the development of boronic acid-based PBP inhibitors involves the repurposing of existing β-lactamase inhibitors or the rational derivatization of their scaffold. This strategy stems from the observation that, despite the availability of numerous acyclic, phenyl, and cyclic boronic acids with nanomolar or sub-nanomolar potency against SBLs and MBLs, their potential as PBP inhibitors remains largely unexplored. Surprisingly, compound **9**, a bicyclic boronic acid originally developed as a BLI, showed exceptional potency against the LMM *EC* PBP5 (dacA), with an IC_50_ of 1.6 nM (Figure 8) [25]. Notably, no inhibition was observed against *Pseudomonas aeruginosa* (*PA*) PBP3, and all other benzoxaborinine compounds tested in the study showed no activity against either *EC* PBP5 or *PA* PBP3. Follow-up studies investigated whether the rigid architecture of cyclic and phenylboronic acids could facilitate selectivity toward bacterial PBPs and β-lactamase targets over proteases, particularly in comparison with more flexible acyclic scaffolds. To achieve this, Newman et al. employed high-throughput protein crystallography to assess the binding interactions of a boron-enriched fragment library with *PA* PBP3 [45]. Rather than potent inhibitors, structural analyses revealed striking differences in the binding modes of three classes of boronic acid inhibitors: bicyclic boronates (e.g., benzoxaboroles **10** and **11**), monocyclic boronates (e.g., compound **12**), and phenylboronic acids (e.g., compound **13**). Among the synthesized compounds, benzoxaboroles **10** and **11** demonstrated the most promising inhibitory activity against *PA* PBP3, with *K*_i_ values of 74 and 78 μM, respectively. In contrast, the monocyclic boronic acid **12** (vaborbactam, the only clinically approved SBL inhibitor) and the phenylboronic acid derivative **13** showed minimal to no inhibitory effect.

**Figure 9:**
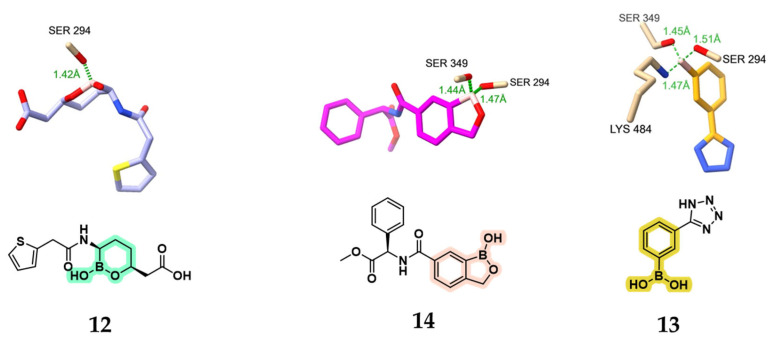
Boronates in complexes with *PA* PBP3. **Left**: vaborbactam (**12**, light purple), exhibiting a mono-covalent mode (PDB: 7AUH). **Middle:** benzoxazole derivative **14** (in fuchsia) displaying a di-covalent binding mode (PDB: 7AU0). **Right**: phenylboronic acid derivative **13** (in yellow), showing a tri-covalent binding mode (PDB: 7ATM).

Despite forming extensive binding interactions within the active site, many of the boronic acid inhibitors displayed poor overall affinity, indicating that binding per se does not necessarily correlate with high potency. Delving into more details, it is pivotal to understand boronic acids can engage PBPs through three distinct covalent binding modes (Figure 9): (a) mono-covalent, involving a single serine residue (compound **12**); (b) di-covalent, engaging two serine residues (compound **14**); and (c) tri-covalent, involving two serine residues and one lysine residue (compound **13**).

Nevertheless, the existence of the tri-covalent mode remains controversial. Newman et al. suggested that this observation in crystallographic structures may be an artifact, noting that some boronate–PBP complexes showed no corresponding inhibitory activity [45]. Still, structures of methylboronic acids (discussed in Section 2.1.3) have been solved in both mono- and tri-covalent forms, indicating the potential for multiple binding configurations. Zervosen et al. proposed a biphasic kinetic model to explain this behavior, where an initial fast mono-covalent complex forms with the serine hydroxyl, followed by a slower secondary step involving a second serine and a lysine residue, ultimately resulting in a tri-covalent adduct [46]. This mechanism aligns with the reversible nature of boronic acid inhibition [47,48]. Although there are no satisfactory proofs and further studies are needed to determine the possibility of multiple binding modes of boronic acids, these studies give potential scenarios to target PBPs, taking advantage of the catalytic residues, namely, the two serines and lysine. Importantly, Zervosen and co-workers attempted to validate the presence of tri-covalent adducts using mass spectrometry (MS). No such species was detected, which the authors attributed to the rapid dissociation of the complex under MS conditions. This highlights a central difficulty in resolving the controversy: while crystallographic and kinetic data suggest that multiple binding modes may be possible, solution-state confirmation remains elusive. An additional confounding factor lies in the intrinsically reversible kinetics of boronic acid–protein interactions, which may obscure detection outside of a crystalline environment. Overall, the current evidence remains inconclusive. Further solution-state investigations are needed to fully establish whether tri-covalent binding is a genuine mechanistic feature or a crystallographic artifact.

**Figure 10:**
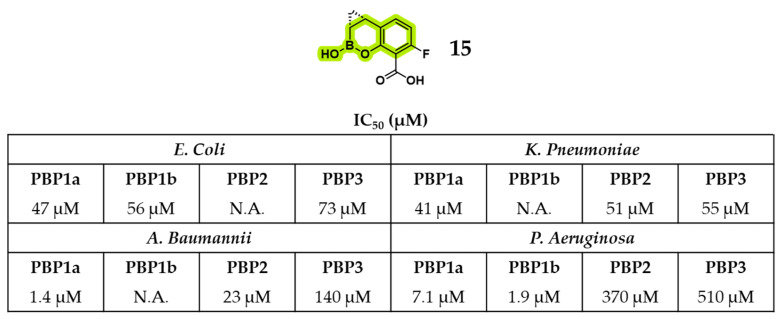
Xeruborbactam (**15**) structure and relative binding activity for four different PBPs of 4 Gram-negative pathogens. In green is highlighted the benzoxaborinine scaffold.

Another benzoxaborinine, the phase I candidate xeruborbactam [49] (compound **15**, formerly QPX7728), exhibited notable intrinsic antibacterial activity in addition to its established role as a BLI. An exhaustive report by Sun et al. demonstrated direct inhibition of both Gram-negative and Gram-positive bacteria, with effective concentrations exceeding those required for β-lactamase inhibition [49]. Importantly, compound **15** enhanced the efficacy of various BLAs even in bacterial strains lacking BL enzymes, underscoring a mechanism of action independent of enzyme inhibition. Its antibacterial effect has been associated with binding to PBPs, and the resulting morphological alterations in *Klebsiella pneumoniae* (*KP*) and *P. aeruginosa* (*PA*) closely resembled those induced by meropenem, suggesting functional disruption of multiple PBPs. Thus, the mode of action of compound **15** has been reported to be consistent with direct interference in cell wall biogenesis. This has also been confirmed by its relative binding activity to HMM PBPs of four different Gram-negative bacteria (Figure 10), including some encouraging IC_50_ values displayed against *Acinetobacter baumanii* (*AC*) PBP1a (IC_50_ = 1.4 μM) and *PA* PBP1b (IC_50_ = 1.9 μM) [49].

**Figure 11:**
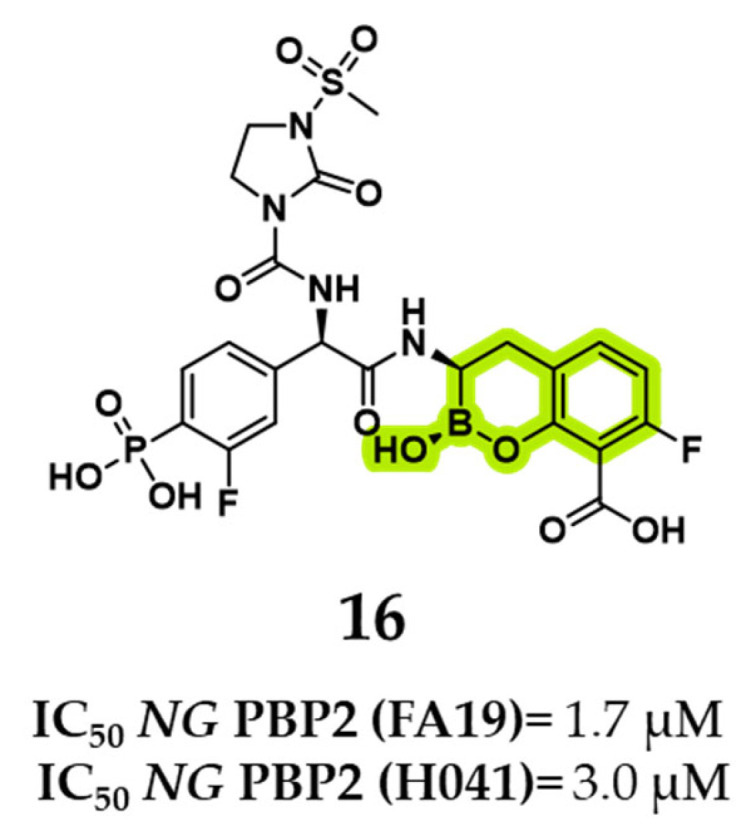
Compound **16**’s structure and relative binding activities for *NG* PBP2 variants. In green is highlighted the benzoxaborinine scaffold.

Venatorx Pharmaceuticals recently developed a promising benzoxaborinine derivative, compound **16** (VNRX-14079), which exhibits potent standalone antibacterial activity, favorable safety and selectivity profiles, and excellent in vivo efficacy [50]. The compound was designed using the benzoxaborinine core scaffold, previously employed by Venatorx in the development of BLIs now in clinical trials, such as taniborbactam and ledaborbactam, and features a cefoperazone-derived side chain. Through SARs optimization, modifications to the amide side chain led to the introduction of a phosphonate moiety, yielding a highly potent PBP2-targeting inhibitor active against *N. gonorrhoeae*, including strains harboring mosaic penA alleles associated with cephalosporin resistance. Compound **16** demonstrated IC_50_ values of 1.7 µM against wild-type *NG* PBP2 (FA19) and 3 µM against the resistant H041 mosaic variant (Figure 11). Notably, compound **16** induces conformational rearrangements in the β3–β4 loop (*NG* PBP2), an essential region implicated in cephalosporin resistance, thereby enhancing engagement with PBP2 even in resistant strains. In microbiological assays, it showed potent bactericidal activity against both mosaic and non-mosaic PBP2-expressing strains, achieving minimum inhibitory concentrations as low as 0.06 µg/mL, surpassing the efficacy of ceftriaxone. Additionally, compound **16** retained robust activity against a diverse panel of 44 clinical *N. gonorrhoeae* isolates, with MIC_50_ and MIC_90_ values of 0.06 and 0.12 µg/mL, respectively. Time-kill studies confirmed rapid bacterial clearance, and no resistance development was observed at concentrations up to 16× MIC [50].

This subsection highlights the potential of repurposing boronic acid-based β-lactamase inhibitors or deriving new PBP inhibitors from BLI scaffolds. Although PBPs and SBLs share significant structural and mechanistic homology, key differences in their active site architecture significantly impact inhibitor efficacy. In particular, many boronic acid inhibitors, while highly potent against SBLs, exhibit only weak or transient inhibition of PBPs [51]. This discrepancy underscores the need for additional structural features tailored to the unique topology and dynamics of PBP active sites. The reversible covalent interaction between the boron atom and the catalytic serine is facilitated by the low energy barrier between sp^2^ and sp^3^ hybridization states [45]. While this feature contributes to the favorable binding kinetics observed in SBL inhibition (e.g., vaborbactam), it may compromise binding stability in PBPs unless reinforced by specific, well-oriented molecular contacts. Thus, despite encouraging advances, boronic acid inhibitors remain less optimized for PBPs than for SBLs, with suboptimal potency and limited selectivity still posing major challenges to therapeutic development. Promising examples include xeruborbactam (**15**) and VNRX-14079 (**16**), which demonstrate direct antibacterial activity, potent PBP binding, and in vivo efficacy. These findings emphasize the structural adaptability of boronic acids, which can be chemically tailored to engage the conformationally dynamic active sites of PBPs through diverse binding modes.

#### 2.1.3. Novel Boron-Based Molecules as PBP Inhibitors

**Figure 12:**
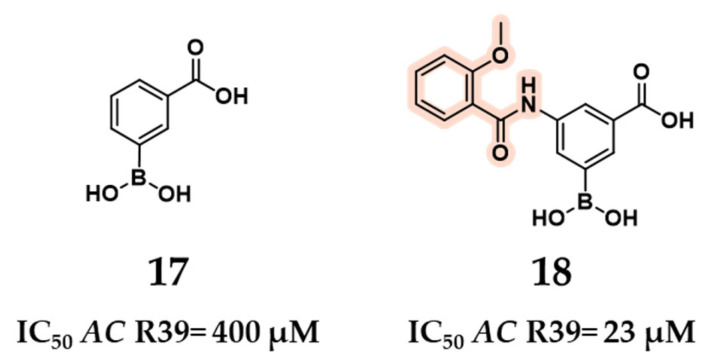
Compounds **17** and **18**’s (modification introduced in pink) structures and relative binding activities for *AC* R39.

In 2009, Inglis et al. identified the 3-(dihydroxyboryl)benzoic acid scaffold (compound **17**) as a promising starting point for the development of inhibitors targeting the d,d-carboxypeptidase *AC* R39 [52]. To support the design of inhibitors for *SP* HMM PBPs, such as PBP1b, PBP2x^R6, and the penicillin-resistant PBP2x^5204, *AC* R39 was employed as a mechanistic and structural model to screen a set of commercially available boronic acids for initial activity. The parent compound **17** exhibited an IC_50_ of 400 µM against *AC* R39. Guided by docking studies, the design was optimized through substitution at the 5-position of the phenylboronic acid ring, introducing a derivatized benzamido group to yield 3-(dihydroxyboryl)-5-(2-methoxybenzamido) benzoic acid (compound **18**). This modification significantly improved potency, with compound **18** showing an IC_50_ of 23 µM against R39 (Figure 12). The rationale for introducing a derivatized benzamido group was based on the expectation that the boronic acid moiety would form a covalent bond with the catalytic serine, while the carboxyl group would engage the same residues observed in the nitrocefin-bound crystal structure. To exploit these predictable anchoring interactions, the benzamido substituent was designed to mimic the C7 side chain of nitrocefin, thereby enhancing interactions with the adjacent subpocket [52].

**Figure 13:**
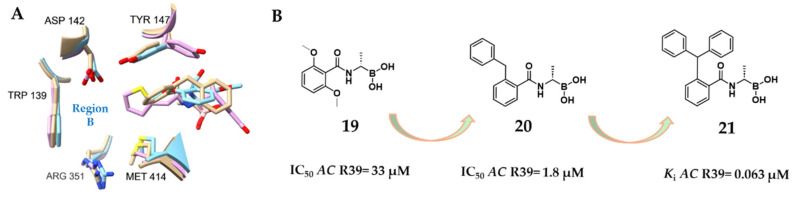
(**A**) Superimposition of the structures of **19** (light blue, PDB: 2XLN), **20** (light brown, PDB: 2XK1), and nitrocefin (light pink, PDB: 1W8Y) in complexes with *AC* R39. (**B**). Evolution of alkyl boronic acids from model compound **19** to compound **21** as potent *AC* R39 inhibitors.

Inglis’s work highlighted the potential of targeting a hydrophobic region within the *AC* R39 active site, flanked by Tyr147 and Trp139. Building on this, Woon et al. investigated the subpocket occupied by the nitrocefin side chain, referring to it as “region B” (Figure 13A). Using the crystal structure of the acyl–enzyme complex between *AC* R39 and nitrocefin as a structural reference, they adopted a multidisciplinary strategy to explore the inhibition of the d,d-carboxypeptidase R39 by various transition state analogs, including trifluoromethyl ketones, phosphonates, and boronic acids [40]. From this campaign, two initial boronic acid-based hits were identified, including compound **19**, which exhibited an IC_50_ value of 33 μM for *AC* R39. Structural analysis revealed that the phenyl ring of compound **19** engages in π-stacking interactions with Tyr147, offering a starting point for optimization. Computational modeling and crystallographic refinement guided the design of compound **20**, featuring a benzyl group introduced in the *o*-position. This substitution exploited the pocket tolerance for lipophilic substituents, resulting in a nearly 20-fold improvement in potency (IC_50_ = 1.8 μM for R39; SPROUT score = −7.04). Interestingly, the benzyl moiety of compound **20** extended into a different pocket, defined by residues Ser49, Glu178, Phe297, and Ser298, while the benzamido group continued to maintain π-stacking with Tyr147. The structural insights obtained from the R39:**20** complex enabled the design of compound **21**, which incorporated a triphenylmethyl substituent appended to the aminoacyl side chain of the boronic acid core. This final derivative achieved the highest potency in the series, with an IC_50_ value of 63 nM (SPROUT score = −7.96) against the LMM R39 (Figure 13B). Collectively, these studies revealed the importance of targeting specific key sub-pockets, including the *AC* R39 region B, for achieving high-affinity binding in boronic acid inhibitors [40].

**Figure 14:**
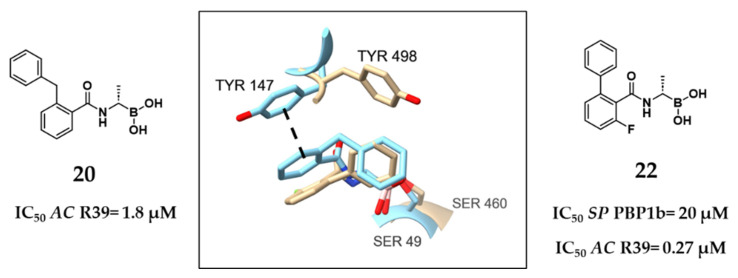
Superimposition of the structures of **20** (light blue, *AC* R39, PDB: 2XLN) and **22** (light brown, *SP* PBP1b, PDB: 2Y2P). π-stacking interaction represented with a black dashed line [53].

Still focusing on Gram-positive pathogens, Contreras-Martel et al. employed *S. pneumoniae* PBP1b, an HMM PBP, as a model enzyme to guide the discovery of novel inhibitors using a crystallography-driven approach [53]. The initial findings showed that aryl boronates were poorly suited for this target, leading to the exploration of alkyl-substituted acetamido boronic acids. These compounds were successfully co-crystallized with PBP1b, revealing distinct side chain binding modes and providing structural insights into sub-pocket engagement. Among them, compound **22** emerged as the most potent, displaying an IC_50_ of 20 μM against *SP* PBP1b and exhibiting direct antibacterial activity against several clinically relevant Gram-positive strains. Interestingly, compound **22** was also tested against the *AC* R39, where it showed much greater potency (IC_50_ = 0.27 μM). Structural comparison between compounds **20** and **22** provides critical insight into this variability (Figure 14). While both compounds share similar positioning of their boronic acid warhead relative to their respective catalytic serine residues (e.g., Ser49 in *AC* R39 and Ser460 in *SP* PBP1b), their surrounding binding pockets differ substantially in their 3D architecture. For example, in the compound **20**–R39 complex, the phenyl group engages in π-stacking with Tyr147, contributing to strong binding. However, in the *SP* PBP1b–compound **22** complex, Tyr147 is rotated nearly 90°, abolishing this interaction and leading to a marked reduction in potency. This observation reinforces a broader principle: conserved catalytic residues alone do not guarantee effective inhibition. Instead, subtle conformational differences in the surrounding pocket environment can profoundly affect binding affinity and inhibitory activity. Together with earlier findings on peptidomimetic boronic acids, these results emphasize that future PBP inhibitor design should prioritize structural dynamics and local architecture around the active site, particularly when comparing PBPs across different bacterial species [53].

**Figure 15:**
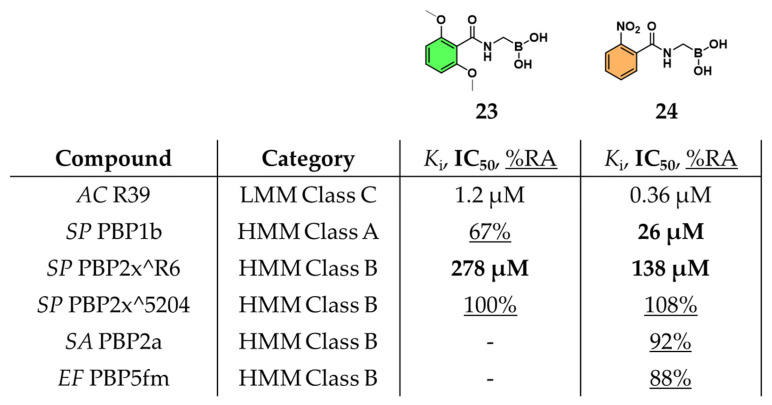
Amidomethylboronic acids **23** and **24** and affinity for different PBPs. *K*_i_ data are indicated in normal font, IC_50_ is in bold, and data indicating percentage of residual activity (%RA) are underlined.

Subsequently, Zervosen et al. explored the inhibitory potential of various amidomethylboronic acids by soaking them into pre-formed crystals of the model *AC* R39. Upon solving the crystal structures and measuring their binding affinities, they unexpectedly observed the formation of tri-covalent boronate adducts within the R39 active site. Given the structural resemblance between the TP domain of PBPs and class A BLs, the side chain of compound **23**, structurally resembling that of methicillin, was identified as a key determinant of activity. In the same article, another compound, 2-nitrobenzamidomethylboronic acid (**24**), was also reported to display a potent *K*_i_ of 0.36 μM [39]. To further explore the role of β-lactam side chains in PBP inhibition, a series of acylaminomethylboronic acids bearing side chains derived from various penam antibiotics were synthesized [47]. Among these, the previously reported compound **24**, emerged as a broad-spectrum inhibitor, displaying potent activity across class A (PBP1b, IC_50_ = 26 µM), class B (PBP2x^R6, IC_50_ = 138 µM), and class C (R39, data shown above) PBPs from penicillin-sensitive strains (Figure 15). These results underscored the significant influence of the side chain structure on inhibitory potency, paralleling observations made in kinetic studies of penams with variable side chains. Despite their promising enzymatic activity, compounds **23** and **24** lacked antibacterial efficacy when evaluated against a panel of bacterial strains, suggesting a possible disconnect between in vitro PBP inhibition and cellular activity.

**Figure 16:**
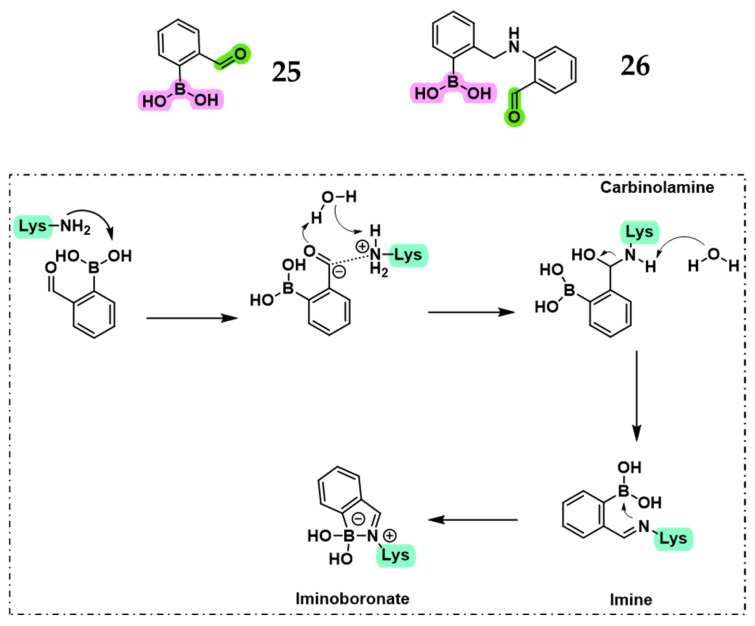
Compounds **25** and **26**, with potential dual anchoring, and predicted mechanism of action (dotted box). The boronic acid moiety is highlighted in fuchsia, the carbonyl in green, and the lysine in light green.

Recently, Kollar et al. investigated whether inhibition of *SP* PBP1b could be redirected from the canonical catalytic serine to the adjacent lysine in the Ser-Lys catalytic dyad [48]. A series of phenylboronic acids, including compounds **25** and **26**, were designed with appropriately positioned carbonyl groups to enable imine formation with lysine, stabilized by dative N → B bonds, resulting in iminoboronate or diazaborine adducts with potential for covalent PBP inhibition (Figure 16). Targeting lysine is mechanistically appealing, as it may circumvent β-lactamase-mediated resistance, which relies on hydrolysis of serine-linked intermediates. Supporting this shift in strategy, both computational and experimental findings indicated that conventional serine-targeted boronic acids offer insufficient free energy gain for effective PBP1b binding. In contrast, lysine-directed inhibition using 2-formylphenylboronic acid derivatives was more promising, with QM/MM simulations providing mechanistic support for covalent modification of Lys463 [45,48,53]. However, compounds **25** and **26** showed weak inhibition of the HMM *SP* PBP1b (IC_50_ = 119 µM for **25**; ~50% residual activity at 500 µM for **26**). Nevertheless, the dual-anchoring strategy, where the boronic acid targets the catalytic serine and a carbonyl group engages the lysine residue, remains a promising approach, particularly with further structural optimization.

## 3. Conclusions

PBPs remain essential antibacterial targets due to their exclusive occurrence in bacterial cells. Although the long-standing clinical success of β-lactam antibiotics, the continued emergence of resistance severely threatens their efficacy, underscoring the urgent need for non-β-lactam chemotypes. Boronic acid inhibitors, originally developed as mechanistic probes, have re-emerged as promising candidates for PBP inhibition. Their value as versatile electrophilic warheads has been successfully exploited against serine hydrolases, in particular, for the development of potent β-lactamase inhibitors. Despite these advances, major challenges persist. Limited cellular efficacy, fragmented assay coverage across bacterial species, and inconsistent outcomes have contributed to a medicinal chemistry bias toward β-lactamase targets, where BAIs consistently deliver nanomolar inhibition.

Repurposing known boron-based β-lactamase inhibitors for targeting PBPs often faces several challenges, as structural and dynamic divergences between the two enzyme families limit translation. A representative example is class A β-lactamases, which present solvent-exposed, flexible pockets (i.e., Ω-loop mobility), while PBP active sites are deeper, more conformationally restricted clefts optimized for recognition of stem peptides. At the same time, PBPs can display adaptive malleability, as exemplified by PBP2a in MRSA, underscoring the importance of considering conformational transitions during inhibitor design. Moreover, whereas β-lactams form long-lived acyl–enzyme complexes with PBPs, boronate adducts are reversible and often transient unless reinforced by stabilizing interactions. Encouragingly, recent advances point to viable solutions. Cyclic boronates, such as compounds **15** (xeruborbactam) and **16** (VNRX-14079), demonstrate how optimization of established BLIs scaffolds can be a productive strategy and deliver intrinsic antibacterial activity by targeting essential PBPs.

Looking forward, this perspective aims to define structure-guided design, dual-anchoring strategies, and systematic SAR campaigns across representative HMM PBPs as the key approaches to adopt for the development of the next generation of inhibitors. Crucially, these efforts must be coupled with improved assay standardization across bacterial species and medicinal chemistry approaches aimed at enhancing cell permeability and pharmacokinetics. These findings support a necessary paradigm shift: rather than designing BAIs for β-lactamases and retroactively testing them against PBPs, drug discovery should instead prioritize PBPs as primary targets, with inhibitors specifically optimized for their structural context.

In summary, while BAIs have not yet matched the potency of β-lactams against PBPs, their unique chemistry, coupled with emerging scaffolds and deeper structural insights, provides a strong foundation: there is a bright future for BAIs as inhibitors of PBPs. A renewed, forward-looking focus on PBPs as primary rather than secondary targets could ultimately unlock the therapeutic potential of boronic acid scaffolds in the fight against antibiotic resistance.

## Data Availability

No new data were created for this perspective.
